# Training on PD-L1 scoring in non-small cell lung cancer with high intra- and inter-reader agreement: results of a worldwide microscopic/digital image-based training of 751 pathologists

**DOI:** 10.3389/bjbs.2026.16477

**Published:** 2026-06-08

**Authors:** Gudrun Bänfer, Rolf Diezko, Rabea Oberthür, George L. Kumar, Josef Rüschoff, Hans-Ulrich Schildhaus, Bharat Jasani

**Affiliations:** 1 Department Biomarker Academy, Discovery Life Sciences Services GmbH (Formerly Targos Molecular Pathology GmbH), Kassel, Germany; 2 Discovery Life Sciences Services GmbH (Formerly Targos Molecular Pathology GmbH), Kassel, Germany; 3 Department of Pathology, Discovery Life Sciences Services GmbH (Formerly Targos Molecular Pathology GmbH), Kassel, Germany; 4 Institute of Pathology Nordhessen, Kassel, Germany; 5 School of Medicine, Cardiff University, Cardiff, United Kingdom

**Keywords:** biomarker training, immunotherapy, non-small cell lung cancer (NSCLC), pathologist training, PD-L1, TPS, Inter-reader, Intra-reader

## Abstract

Semi-quantitative scoring of PD-L1 expression on tumor cells (TCs) and/or infiltrating immune cells (ICs) is complex and requires expert pathologist training to reduce inter- and intra-reader variability. We conducted a 1- or 2-day training of 751 pathologists world-wide from 63 countries over a period of 2 years (2016 and 2017). Pathologists read microscopic slides or fully digitized slides stained with PD-L1 immunohistochemistry 22C3 pharmDx assay with expression levels enriched around the clinically relevant cutoffs of 1% and 50% in non-small cell lung cancer (NSCLC). The overall inter-reader agreement (OPA) for PD-L1-stained NSCLC was 95.6% for TPS ≥1% and 87.3% for TPS ≥50% cut-off. The corresponding intra-reader agreement was 95.9% for the ≥1% and 91.4% for the ≥50% cut-off. The inter-reader negative percent agreement (NPA) for TPS ≥1% was 85.6% and 91.9% for the TPS ≥50% cut-off, and the positive percent agreement (PPA) was 97.6% (TPS ≥1%) and 81.0% (TPS ≥50%). The observed high inter- and intra-reader agreements are promising given the difficulty of reproducible scoring tumor cells with heterogeneous distribution of PD-L1 staining combined with the varied professional training, expertise, and experience of the participants. The results may reflect the utility of the expert led standardized protocol used to train pathologists for scoring PD-L1 staining in NSCLC specimens. The digital image-led training approach has the additional advantage of providing a computer-assisted scoring system and allows for remote training of pathologists worldwide.

## Introduction

Lung cancer is the leading cause of cancer-related deaths globally in both men and women, with an estimated of 1.8 million people diagnosed and 1.6 million deaths. An estimated 236,740 people are likely to be diagnosed with lung cancer in 2022 in the U.S. with an expected death rate of 130,200 per annum [1].

Non-small cell lung cancer (NSCLC) is the predominant type, representing approximately 85% of cases with poor prognosis for patients with advanced/metastatic disease [2] or Platinum-based chemotherapy remains the first-line treatment of choice for advanced NSCLC patients who do not harbor activating driver mutations but the addition of immune checkpoint blockade has improved the treatment of NSCLC, making long-term survival possible [3].

The programmed cell death 1 (PD-1) receptor and one of its two ligands, programmed death-ligand 1 (PD-L1), have been found to be effective immune checkpoint targets for both second and first line treatment [4–9].

The US Food and Drug Administration (FDA) and European Medicines Agency (EMA) have approved first- and second-line treatments using a PD-1/PD-L1 blocking antibodies for NSCLC. The percentage of PD-L1 stained cells (i.e., tumor proportion score (TPS) in NSCLC tumors is used to determine the PD-L1 expression level before treatment with a specific drug such as pembrolizumab.

Pembrolizumab, is a humanized monoclonal IgG4-κ isotype, anti-PD-1 antibody originally approved by the FDA in 2015 as second-line therapy for NSCLC together with the PD-L1 IHC 22C3 pharmDx companion diagnostic test by Dako (Carpinteria, CA, USA) [5,7,10].

For NSCLC patients with PD-L1 expression in ≥50% of tumor cells, pembrolizumab has been shown confer a superior progression-free survival (PFS) and overall survival (OS) compared with platinum-doublet chemotherapy in the first-line setting. Whilst for PD-L1 expression of 1%–49%, programmed death-1 (PD-1) or PD-L1 inhibition has been shown to be comparable to chemotherapy. In contrast, for patients with negative PD-L1 expression (approximately 50% of all cases), there is currently no proven therapeutic strategy available [11].

In 2016, a TPS ≥1% was added as a cutoff and then in 2019 the pembrolizumab approval was expanded to become a first-line treatment for NSCLC, either as single agent or in combination with other therapy (for further information see FDA.gov). In parallel, the EMA approved pembrolizumab as a first-line monotherapy, referring to a validated biomarker test for PD-L1 expression in NSCLC (TPS 50%), and as second line combination therapy for tumors with and without EGFR or ALK positive mutations (https://www.ema.europa.eu). Patients confirmed with PD-L1-positive disease via IHC have a higher overall response rate to pembrolizumab and improved progression-free and overall survival rates [5, 12].

However, accurate and consistent assessment of the IHC-detectable PD-L1 expression status presents significant challenges and pitfalls for pathologists together with a wide variability in the distribution of PD-L1 scoring. Therefore, necessitating careful training in interpretation and scoring to reduce intra- and inter-reader variability is a helpful tool for pathologists [13–15].

For nearly 15 years, Discovery (formerly Targos) has led the formal training of pathologists worldwide on the interpretation and scoring of human epidermal growth factor receptor 2 (HER2) expression in breast and gastric cancers [16–18].

To meet this need for accurate interpretation and scoring of PD-L1 as a predictive biomarker of NSCLC, pathologists were either invited to Kassel, Germany, for face-to-face training using microscopic slides or sent expert pathologists to different countries to train pathologists using digital scanned images obtained from these same microscopic slides.

Here we describe the results of expert led training on PD-L1 22C3-stained slides and whole slide images derived from these, for scoring TPS in NSCLC, and investigate the accuracy and intra- and inter-reader reproducibility of scoring TPS at 1% and 50% cutoff in training a large number of pathologists across the world.

## Materials and methods

### Training sets – glass slides and digital images

The PD-L1 trainings were performed using glass slides or corresponding whole slide scanned digital images viewed with the image software Aperio Image Scope (Aperio) (Leica Biosystems Nußloch GmbH, Nußloch, Germany). Slides were scanned by Discovery Life Sciences Services GmbH (Kassel, Germany) using Aperio ScanScope XT scanners for use with Aperio (Leica Biosystems Nußloch GmbH, Nußloch, Germany) at ×40 magnification for PD-L1 stained slides and at ×20 magnification for hematoxylin and eosin (H&E) as well as negative control reagent (NCR) stained slides.

H and E, negative control reagent and PD-L1 stained glass slides, respectively, representative of resection tumour specimens of fifty-four NSCLC (adenocarcinomas and squamous carcinoma) cases were obtained from Dako (Carpinteria, CA, USA) for the training. Fourteen of these cases were selected for demonstration purposes, and ten for pretest (two sets of five cases each) and 30 cases for the main test, respectively (Supplementary Table S1).

All immunostaining was performed by Agilent/Dako (Carpinteria, CA, USA) on formalin-fixed paraffin-embedded sections and the specimens were stained for PD-L1 using PD-L1 IHC 22C3 pharmDx (Code SK006, Agilent, Santa Clara, CA, USA) according to the manufacturer’s instructions, including an NCR (monoclonal mouse control IgG antibody) for reagent negative control.

### PD-L1 scoring

The PD-L1 score is the Tumour Proportion Score, TPS, for each case was determined through a consensus amongst a panel of three expert pathologists from Dako who had initially evaluated the cases independently.

The TPS is defined as the percentage of viable tumor cells showing partial or complete membrane staining at any intensity relative to all viable tumor cells present in the sample. Importantly, cytoplasmic staining and stained infiltrating immune cells, normal cells, and necrotic tissue were all excluded from scoring and only tumor specimens containing at least 100 viable tumor cells were included. Specimens were interpreted as being “PD-L1 negative” with TPS <1%, PD-L1, weak positive’ with TPS ≥1% but <50%, and “PD-L1, strong positive” with TPS ≥50% (PD-L1 IHC 22C3 pharmDx interpretation manual, Agilent Technologies, 2021). Cutoff points for positivity were defined according to 1% TPS and 50% TPS thresholds as originally defined by Dako [6].

### Training protocol

Two Discovery pathologists (JR, HUS) were initially trained by Dako/Agilent expert pathologist involving a 2-day expert-led PD-L1 training schedule. Training of a further six Discovery pathologists was then provided by the trained pathologists JR and HUS following the Dako/Agilent’s US FDA-approved interpretation and scoring manual. Systematic trainings for PD-L1 scoring in NSCLC were then globally organized and conducted by individual Dako certified Discovery pathologists with sponsorship from Merck Global (Kenilworth, NJ, US).

### Pathologists training

PD-L1 training was conducted either as a 1-day or 2-day training course. For the 1-day course, a background lecture on how to score followed by an interactive microscope session that included a self-assessment exercise was given. Here participants were given 20 cases. For the 2-day training, an additional 25 cases were given on Day 2, which included 15 cases from day 1. A comparison of the 1-day and 2-day training programs is shown in Supplementary Table S2 and described in further detail below.

The 2-day training was designed to train pathologists to become expert trainers themselves for teaching interpretation and scoring of PD-L1 22C3 stained slides/digital images. This course was referred to as “Train the Trainer” training.

**FIGURE 1:**
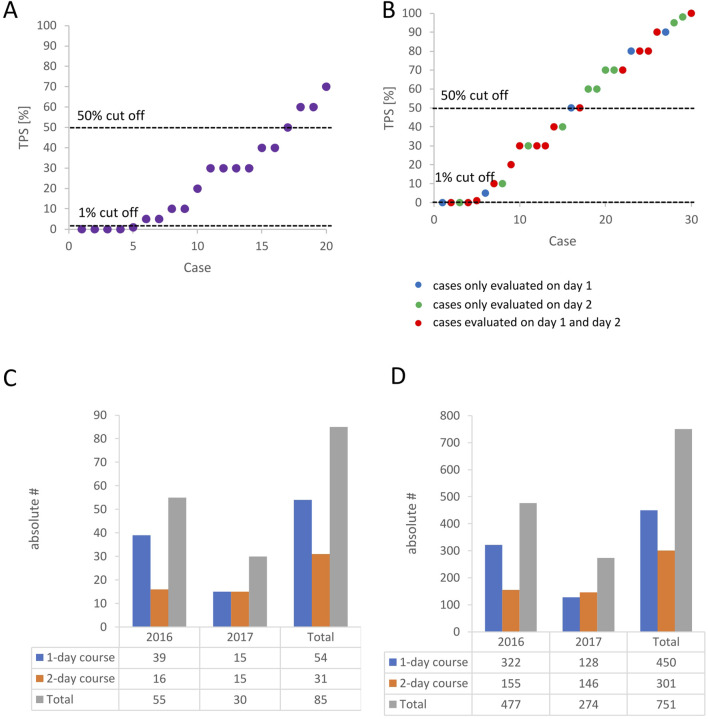
Overview of case distribution and information on completed PD-L1 trainings (2016–2017). **(A)** Case distribution in a 1-day PD-L1 training. **(B)** Case distribution in a 2-day PD-L1 training. Blue cases were only evaluated on day 1, green cases were only evaluated on day 2, and red cases were evaluated on day 1 and day 2. **(C)** Absolute numbers of 1-day and 2-day PD-L1 trainings in 2016 and 2017. **(D)** Absolute numbers of participants attending 1-day and 2-day PD-L1 trainings in 2016 and 2017.

The 2-Day training included a background lecture on PD-L1 biology followed by a detailed presentation of interpretation and scoring of the PD-L1 IHC 22C3 pharmDx in NSCLC with illustrative examples. Following this, the trainer demonstrated slides from 10 to 14 cases, either directly on a multiheaded microscope (Leica Microsystems CMS GmbH, Wetzlar, Germany) or via high resolution images displayed on-screen or projected images using Aperio. The scoring algorithm was reviewed and the value of adopting a systematic approach to interpretation and scoring based on H&E, NCR and PD-L1 stained slides was emphasized. The demonstration comprised cases showing no staining or varying distribution and degrees of weak-to-strong tumor-specific membrane PD-L1 expression. The participants then performed a self-assessment pretest, based on the scoring of five selected cases displaying a range of PD-L1 staining. Following this, the individual case results and any questions raised by the participants were discussed in detail. Participants then proceeded to complete a formal competency assessment (referred to as the Main Test) that included an evaluation of 20 cases selected to represent different levels of tumor associated PD-L1 expression (Figure 1A).

On the second day, a second formal competency assessment was conducted to further evaluate inter-reader variability and assess intra-reader reproducibility in scoring tumor-associated PD-L1 expression. Pathologists scored 25 NSCLC cases that included 15 cases taken from the day 1 test, masked with different identifiers (for assessment of intra-reader concordance) along with 10 new cases not previously evaluated by the participants (as additional cases for inter-reader concordance) (see protocol described in Figure 1B). At the end of the Day 2 test, the intra- and inter-reader concordance results were calculated and presented to participants, and any difficult or strongly discrepant cases were discussed in detail. Each participant received a certificate of completion for the 2-day training if they met a concordance rate of at least 85% in all four categories (1% cutoff – intra-reader agreement, 1% cutoff – inter-reader agreement, 50% cutoff – intra-reader agreement, 50% cutoff – inter-reader agreement). The participants who failed to achieve 85% in all four categories were given a certificate for participation.

The 1-day training was designed to compare only the inter-reader agreement between participating pathologists and the expert derived consensus scores for a total of 20 cases. Otherwise, the training was identical to the 2-Day training described above.

### Statistical analysis

Statistical analyses were conducted using Microsoft Excel. According to the consensus TPS provided by Agilent for each case, cases were defined as positive or negative in concordance to the 1% and 50% cutoff points. The inter- and intra-reader agreement was determined using overall percentage agreement (OPA), positive percent agreement (PPA), negative percent agreement (NPA) and Cohens ĸ coefficient. The 95% confidence interval (CI) was calculated for all computations. Inter-reader concordance was analyzed for 1-day and 2-day trainings, whereas the intra-reader concordance was calculated for 2-day training only. Since test cases were evaluated by a different number of pathologists, OPA, PPA and NPA differ from the mean value used for the determination of the 95% CI.

## Results

### Participant background

**FIGURE 2:**
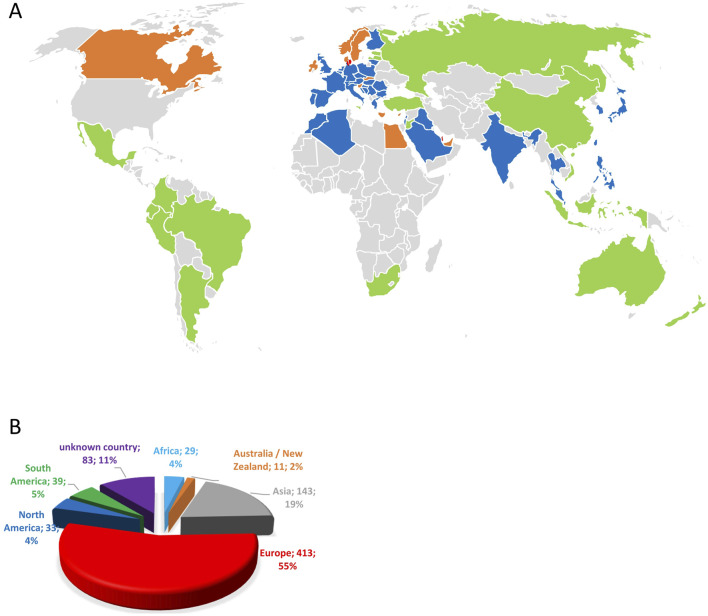
Training Courses Overview. **(A)** Origin of participants and **(B)** country-wide locations of PD-L1 trainings.

Altogether a total of 751 participants were trained scoring NSCLC across 85 training sessions over a period of 2 years. In the first year, 322 participants were trained in 1-day training sessions (n = 39) and 155 participants in 2-day training sessions (n = 16). In the second year, 128 participants attended the 1-day sessions (n = 15) and 146 pathologists participated in 2-day training sessions (n = 15) (Figures 1C,D). The PD-L1 training courses were attended by participants worldwide: in the first year, 477 participants from 58 countries attended the training courses, and in the second year, 48 countries were represented by attending participants (n = 274). Most of the participants were from Europe (55%), followed by participants from Asia (19%), South America (5%), Canada (4%), Africa (4%), and Australia/New Zealand (2%). Approximately 11% of the participants could not be assigned to a country or a continent since this information was not made available (Figure 2A).

The majority of these courses, roughly 70%, were performed in Europe (Figure 2B).

### PD-L1 expression levels among training cases

**FIGURE 3:**
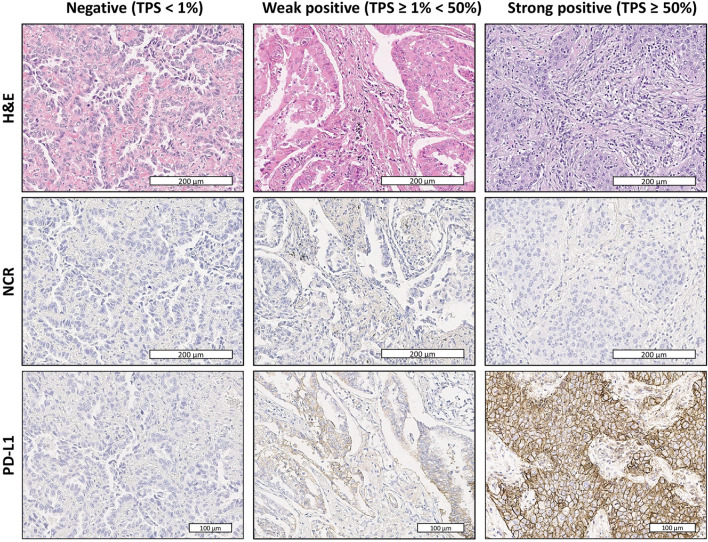
Concordant cases. Example images for PD-L1 negative (TPS <1%), PD-L1 weak positive (TPS ≥1% < 50%), and PD-L1 strong positive (TPS ≥50%) cases. For each case the H&E, NCR, and PD-L1 staining is shown. Scale bar for H&E and NCR staining is 200 μm, for PD-L1 staining 100 µm.

The test case distribution corresponding to the TPS expressed as a % for the 1-day and 2-day training courses are shown in Figures 1A,B, respectively, and in Supplementary Table S2. Among the 20 test cases for the 1-day training, 20% had a TPS <1%, 60% displayed a TPS ≥1% and <50%, and 20% a TPS ≥50%. Among the 30 test cases of a 2-day training, 13.3% had a TPS <1%, 36.7% a TPS ≥1% and <50%, and 50% a TPS ≥50%. Examples of a PD-L1 negative case (TPS <1%), a PD-L1 weak positive case (TPS ≥1% and <50%) and a PD-L1 strong positive case (TPS ≥50%) are shown in Figure 3. All test cases in the 1-day training sessions were also assessed during the 2-day training; however, the 1-day training test set used for the 2-day course lacked “clear” positive cases with TPS ≥70%.

### Competency and success rates associated with training formats and platforms

**FIGURE 4:**
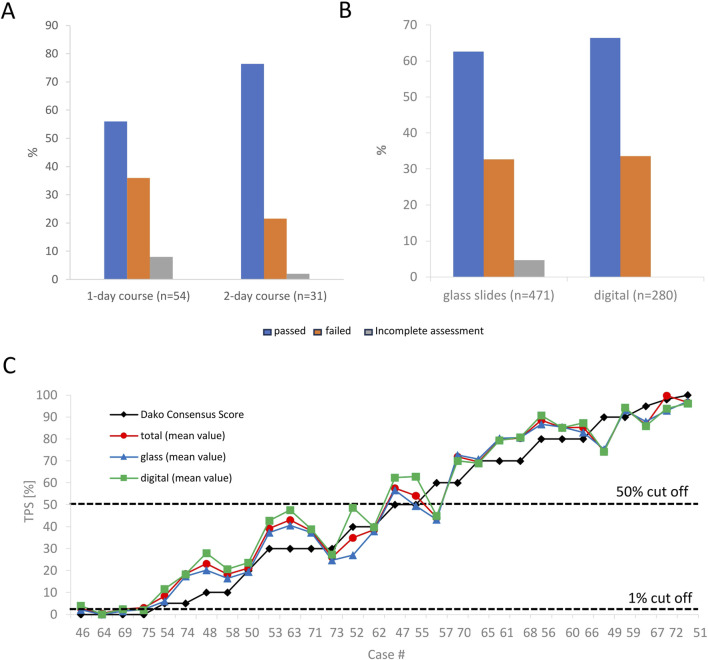
Success rates according to format and platform. Competency success rates associated with **(A)** training formats and **(B)** platforms. **(C)** Total case distribution with consensus scores and participants mean value scores for glass slides and digital images.

Altogether, 64% (n = 482) of all participants (n = 751) attending 1-day and 2-day trainings passed, while 30% (n = 227) of the participants did not meet the 85% concordance criteria. Approximately 6% (n = 42) represent an incomplete assessment (data not shown). Separate analysis of the training participant pass rates demonstrates that 56% (n = 252) passed in the 1-day trainings and 76% (n = 230) passed in the 2-day trainings. There was a participant failure rate of 36% (n = 162/450) in the 1-day training and nearly 22% (n = 65/301) in the 2-day training (Figure 4A).

In 2016, 38 training sessions with 304 participants were performed using glass slides, whereas only 173 participants across 17 training sessions used digitized slides. In 2017, 167 participants across 19 training sessions scored cases using glass slides, while 107 participants across 11 training sessions evaluated digital images (Figure 4B). In total, 57 (67%) trainings with 471 (63%) attending participants using glass slides have been performed in 2016 and 2017, while in only 28 (33%) trainings with 280 (37%) participants used digitized slides (data not shown).

Notably, there was no difference in the pass rate of the participants between the used of glass or digitized slides evaluation platforms; the pass rate was 62.6% with glass slides and 66.4% with digital-based (Figure 4B). The mean TPS scoring of the attending pathologists was compared with the consensus scores defined by three Dako/Agilent expert pathologists for the 30 individual cases (digital images or slides) and in shown in Figure 4C. Altogether, the mean TPS values of glass slides and digital images were highly similar, with only two cases (52 and 55) demonstrating differing mean values. For case 52, the mean values for glass slides (mean TPS = 27.6%) and digital images (mean TPS = 45.3%) were ≥1% and <50% TPS, therefore the outcome regarding the cutoff points was the same. Similar results were observed for case 55, where the mean TPS for glass slides was 50.3% and for digital images TPS was 62.2%. Interestingly, for case 57, the consensus score given by Dako (TPS = 60%) strongly deviates from the mean TPS value of all attending pathologists (total mean TPS = 44.6%). Almost half of all participating pathologists (48.2%) have not met the 50% cutoff point.

### Overall Inter-reader reproducibility

Each sample assessment (n = 16,214) was compared with the reference score given by Dako/Agilent. For the 1% cutoff point, 15,503 (95.6%) samples were scored concordantly with the consensus score, resulting in an OPA of 95.61% (95% CI, 94.4–98.4), while 711 (4.4%) samples were not concordant with the consensus score. Of the non-concordant samples, 2.4% (n = 392) were scored false positive and 2% (n = 319) were scored false negative. The NPA was calculated as 85.6% (95% CI, 81.1–94.6) and the PPA was 97.6% (95% CI, 96.2–99.2). Cohen’s ĸ coefficient was 0.84. For the 50% cutoff point, 14,151 (87.3%) samples were concordant to the consensus score, giving an OPA of 87.28% (95% CI, 84.5–94.3). Of the non-concordant samples at this cutoff, 4.7% (n = 763) were false positive, whereas 8% (n = 1,300) were scored false negative. The NPA for the 50% cutoff point is 91.9% (95% CI, 80–93.7) and the PPA is 81% (95% CI, 85.2–98.6). Cohen’s ĸ coefficient is 0.74 (Table 1).

**TABLE 1:** Inter-reader concordance.

​	Inter-observer concordance	
	1% cut off point (n = 16,214)	50% cut off point (n = 16,214)
OPA [%] (95% CI)	95.6 (94.4–98.4)	87.3 (84.5–94.3)
Concordant to reference score		
No	711 [4.4%]	2,063 [12.7%]
Yes	15,503 [95.6%]	14,151 [87.3%]
Negative - negative^a^	2,328 [14.4%]	8,603 [53.1%]
Negative - positive^b^	392 [2.4%]	763 [4.7%]
Positive – negative^c^	319 [2%]	1,300 [8%]
Positive - positive^d^	13,175 [81.3%]	5,548 [34.2%]
NPA [%] (95% CI)	85.6 [81.1–94.6]	91.9 [80–93.7]
PPA [%] (95% CI)	97.6 [96.2–99.2]	81 [85.2–98.6]
Cohen’s ĸ coefficient	0.84	0.74

Cohen’s ĸ coefficient: ĸ value range from 0 to +1, with 0 indicating no agreement and +1 perfect agreement between the attending pathologists and the consensus score. The strength of Cohen’s ĸ coefficient is defined as no agreement if ĸ ≤ 0.1, poor agreement if 0.1 < ĸ ≤ 0.4, moderate agreement if 0.4 < ĸ ≤ 0.6, substantial agreement if 0.6< ĸ ≤ 0.8, and almost perfect agreement if 0.8< ĸ ≤ 1.

^a^ Negative – negative equals true negative.

^b^ Negative – positive equals false positive.

^c^ Positive – negative equals false negative.

^d^ Positive – positive equals true positive.

Abbreviations: OPA, overall percent agreement; CI, confidence interval; NPA, negative percent agreement; PPA, positive percent agreement.

### Intra-reader reproducibility

Each sample (n = 15) was evaluated twice by the same pathologist to determine the intra-reader reproducibility, resulting in a total of n = 4,441 assessments. For the 1% cutoff point, 4,260 (95.9%) samples were scored concordantly in both assessments by the same pathologist, resulting in an OPA of 95.9% (95% CI, 93.3–98.8), while 181 (4.1%) samples were not scored concordantly between day 1 and day 2. Almost 1.6% (n = 73) of the non-concordant samples displayed a shift from negative to positive whereas 2.4% (n = 107) of the samples showed an adjustment from positive to negative. Cohen’s ĸ coefficient is 0.87. For the 50% cutoff, 4,061 (91.4%) samples were evaluated in concordance on day 1 and day 2, leading to an OPA of 91.4% (95% CI, 85.0–97.0). However, 8.6% (n = 380) of the sample results were not reproduced on the second day, with 171 (3.9%) samples scored as negative during the first assessment and positive during the second, and 207 (4.7%) samples scored as positive on day 1 and then negative on day 2. Cohen’s ĸ coefficient was 0.83 (Table 2).

**TABLE 2:** Intra-reader concordance.

​	Intra-observer concordance	
​	1% cut off point (n = 4,441)	50% cut off point (n = 4,441)
OPA [%] (95% CI)	95.9 (93.3–98.8)	91.4 (85.0–97.0)
Pairwise evaluation concordant		
No	181 [4.1%]	380 [8.6%]
Yes	4,260 [95.9%]	4,061 [91.4%]
Negative – Negative^*^	786 [17.7%]	2,259 [50.9%]
Negative – Positive^*^	73 [1.6%]	171 [3.9%]
Positive – Negative^*^	107 [2.4%]	207 [4.7%]
Positive – Positive^*^	3,469 [78.1%]	1,798 [40.5%]
Cohen’s ĸ coefficient	0.87	0.83

* Results are presented in the following order: first assessment (on day 1) – second assessment (on day 2).

Abbreviations: OPA, overall percent agreement; CI, confidence interval; NPA, negative percent agreement; PPA, positive percent agreement. Cohen’s ĸ coefficient: ĸ value range from 0 to +1, with 0 indicating no agreement and +1 perfect agreement between the pathologists and the consensus score. The strength of Cohen’s ĸ coefficient is defined as no agreement if ĸ ≤ 0.1, poor agreement if 0.1 < ĸ ≤ 0.4, moderate agreement if 0.4 < ĸ ≤ 0.6, substantial agreement if 0.6< ĸ ≤ 0.8, and almost perfect agreement if 0.8< ĸ ≤ 1.

### Inter-reader reproducibility

**FIGURE 5:**
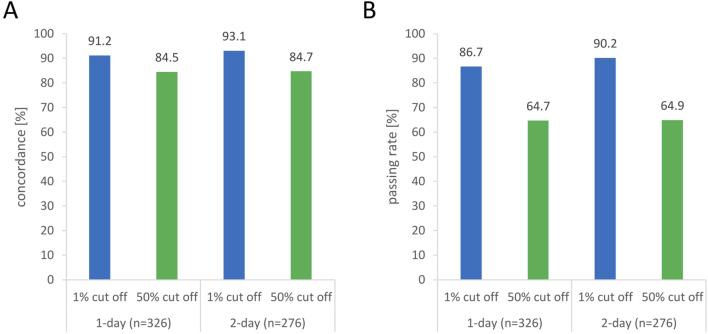
Success rates according to 1% and 50% cutoff points. Concordance and passing rates are similar based on the 20 cases used for 1-day **(A)** and 2-day **(B)** PD-L1 trainings. Concordance and passing rates are lower for the 50% cutoff point in comparison to the 1% cutoff point.

To investigate the influence of the training format, OPA, PPA, NPA and Cohen’s ĸ coefficient were calculated for the cases (n = 20) used between 1-day and 2-day trainings (Figure 5A). Each sample assessment of 1-day (n = 6,460) and 2-day trainings (n = 5,300) was compared with the consensus score given by Dako/Agilent. For the 1-day trainings, 94.6% (n = 6,111) of the cases were evaluated concordantly, which resulted in an OPA of 91.2% (95% CI, 90.3–92.1) for the 1% cutoff point. For the 50% cutoff point, 5,473 (84.7%) samples were scored concordantly, leading to an OPA of 84.5% (95% CI, 83.6–85.5). The PPA was calculated as 96.1% (95% CI, 93.6–98.5) for the 1% cutoff point and 77.8% (95% CI, 63.1–92.6) for the 50% cutoff point, and the NPA was calculated as 88.8 (95% CI, 82.4–95.2) for the 1% and 87% (95% CI, 80.1–93.9) for the 50% cutoff point. Cohen’s ĸ was 0.83 and 0.61, for the 1% and 50% cutoff points, respectively. For the 2-day trainings, 95% (n = 5,300) of the cases were scored concordantly, which resulted in an OPA of 93.1% (95% CI, 92.2–94) for the 1% cutoff point. Of the 264 (5%) discordant cases, 130 (2.5%) negative cases were scored false positive and 134 (2.5%) cases were evaluated false negative. NPA was 87.7% (95% CI, 81.5–94), PPA was 96.8% (95% CI, 95.1–98.6) and Cohen’s ĸ was 0.85. For the 50% cutoff point, OPA was 84.7% (95% CI, 83.6–85.8) and 4,492 (84.8%) cases were scored concordantly while 808 (15.2%) were scored non-concordantly. Of the 808 (15.2%) discordant cases, 522 (9.9%) were scored false positive and 286 (5.4%) false negative. NPA was 86.9% (95% CI, 80.2–93.5), PPA was 78.4% (95% CI, 65.9–90.9) and Cohen’s ĸ was 0.62 (data not shown).

### Success rates according to 1% and 50% cutoff points

For 1-day trainings the passing rate of the participants was 86.7% for the 1% cutoff point and 64.7% for the 50% cutoff point. For 2-day trainings, the passing rate was 90.2% and 64.9% for the 1% and 50% cutoff points, respectively. This suggests that the 50% cutoff point was more difficult for the pathologists to score correctly than the 1% cutoff point. Furthermore, these results show that the training duration format had no impact on training success (Figure 5B).

### Evaluation of participant feedback

At the end of each training the participants evaluated the session by assigning scores to eight categories: overall fulfillment of expectations, quality of the background lecture, demonstration sessions, self-assessment, interactivity, instructors, information material, and overall organization. Grades were on a scale of 1–5, where 1 = “fully disappointed” and 5 = “fully satisfied.”

**FIGURE 6:**
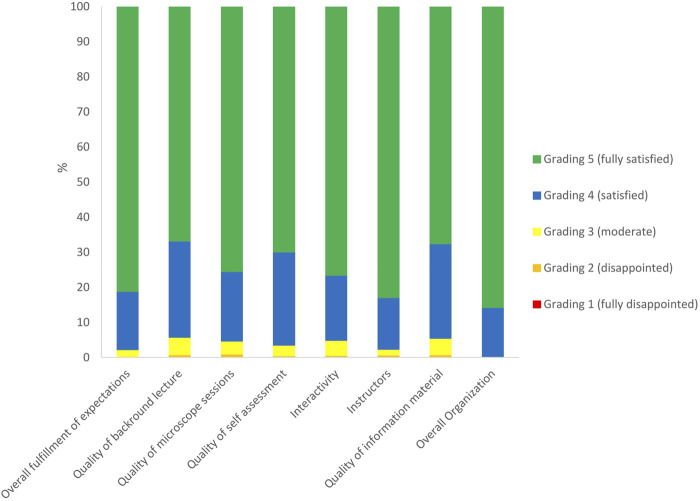
Participant feedback on PD-L1 trainings. Participants gave feedback voluntarily at the end of the PD-L1 trainings. Grading ranged from 1 = “fully disappointed” to 5 = “fully satisfied.”

Altogether, the participants graded the trainings as “very good.” More than 80% of all participants stated that the trainings fulfilled their expectations. The demonstration sessions, self-assessment, interactivity, and overall organization were all graded ‘very good’ by more than 70% of the participants. The background lecture and information material provided were graded as ‘very good’ by 66.9% and 67.6% of the participants, respectively. The instructor category received the highest overall grade, with up to 84% of participants assigning a top score (Figure 6).

## Discussion

The present study relates to the world’s first world-wide systematic training of a large cohort of pathologists using TPS scoring of tumor-associated PD-L1 in NSCLC stained with the FDA-approved PD-L1 IHC 22C3 pharmDx, on either microscope slides or fully digitized images.

Following the introduction of PD-L1 IHC as a companion diagnostic assay for NSCLC and other cancer types, the requirement for expert training has become even more critical, as pathologists are required to score the results at different cutoffs for different therapy indications [19]. Standardized interpretive training, based on an adequate number of samples spanning the full range of PD-L1 IHC staining, is necessary to ensure low inter-observer variability in PD-L1 staining interpretation.

So far at least seven studies, including this one, have been conducted to assess inter-observer variability in assessment of PD-L1 score for NSCLC. The only previous study of pathologist training and scoring assessment of NSCLC samples stained with the companion diagnostic assay method PD-L1 IHC 22C3 pharmDx, involved a two-stage assessment of 60 cases by 10 pathologists, with a 1-h training between the two assessments [20]. Similar to the present study, the scoring was directed solely at the estimation of TPS. The intra- and inter-reader OPA for 1% and 50% thresholds were observed to be 89.7 (95% CI, 85.7–92.6) and 91.3% (95% CI, 87.6–94), and 84.2% (95% CI, 82.8–85.5) and 81.9% (95% CI, 80.4–83.3), respectively, with no significant impact of the 1-h training on the overall outcome.

In the present study, the corresponding intra- and interobserver rates were 95.9 (95% CI, 93.3–98.8) and 91.4 (95% CI, 85.0–97.0) and 95.6 (95% CI, 94.4–98.4) and 87.3 (95% CI 84.5–94.3), respectively, observed for the 2-day training. The inter-reader rates observed both for the 2-Day and 1-Day training for scoring the cases at 1% threshold matched the minimum of >90% concordance rate observed by Williams et al. [21] based on online 1-Day training of 6 expert lung pathologists using Ventana online training module based on image analysis of PD-L1 (SP263) in NSCLC (https://education.ventana.com/mod/page/view.php?id=140). Unlike Williams et al. [21] and Cooper et al. [20] studies, the present study included a 2-Day training with a “wash-out” period of 1 day. For this an inter-observer OPA of 91.2% (95% CI, 90.3–92.1) for the 1% cutoff point and 84.5% (95% CI, 83.6–85.5) for the 50% threshold were observed for the very large cohort of pathologists trained. The inter-reader concordance rates of both the 1-day and 2-day trainings are consistently better than those observed by Cooper et al. [20]. Our results also represent better outcomes than those reported in [22] at the 50% cutoff, in a study that involved 13 expert pathologists analyzing 90 NSCLC cases stained with the 22C3 clinical trial assay, which found an inter-reader concordance of 82.2% (95% CI, 87.3–89.1).

However, there were several outlier cases (n = 7) that showed a significantly lower than average inter-reader agreement. Likely reasons for the higher discrepancies included close proximity of the consensus score to the threshold of 1%; focally distributed, tumor-related PD-L1 staining present in a background of peri-tumoral inflammation; necrosis and a focally distributed PD-L1 positive cluster; and fixation artifacts.

Ignoring these difficult cases, the overall high average success rate of the training described in the present study may be attributed to a combination of the following elements:

1. In person training, i.e., either at Discovery site in Kassel Germany or at a remote site.
2. The PD-L1 IHC 22C3 pharmDx interpretation training from Agilent
3. A standardized training protocol based on the manufacture’s (Agilent) scoring guide for reading PD-L1 staining in tumor samples.
4. Highly competent trainers who had achieved the (Agilent) PD-L1 the training threshold of 85% on both day 1 and day 2.
5. Glass slides or equivalent digitized images scanned that were originally used by an independent panel of expert pathologists to derive the consensus scores.
6. Initial familiarization of the trainees with samples from a dynamic range of tumor-related PD-L1 staining patterns and distribution, followed by a self-assessment test and a critical review of the discrepant results, prior to the competency assessment with the 1-day or 2-day test slides.

It is also noteworthy that we observed equivalent training success regardless of the use of glass slides or digital images. The digital image-led training was originally introduced mainly to overcome the risks and logistical difficulties involved in the transportation of the unique set of training slides to the various training centers around the world. The use of digital images also afforded the possible advantage of reducing inter-reader variability via the use of standardized digital image-based analysis, similar to what was observed in the more reproducible intra- and inter-reader scoring of HER2 expression in breast cancer [23]. Such an approach may help in the design of more robust scoring methods, capable of improving the overall clinical utility of novel immunohistochemical biomarkers in the future.

## Conclusions

The study demonstrates the success and utility of a standardized protocol and program used to train pathologists to an expert level of competency for scoring PD-L1 staining in NSCLC specimens, using either glass slides or whole slide scanned digital images. The consistently high inter- and intra-reader agreements achieved following 1- or 2-day face-to-face training are promising given the complexity of scoring tumor cells which often contained a heterogeneous distribution of PD-L1 staining; the varied background, expertise and experience of the participants; and the use of either glass slides or digital images. These training programs are critical to not only support pathology education to accurately evaluate and score PD-L1 expression in patients with NSCLC for KEYTRUDA eligibility but also help oncologists prescribe the correct drug to the right patient at the right time at the right dose and right duration.

## Summary table

### What is known about this subject

- There is continuing wide variability amongst pathologists in assessment of PD-L1 status in non-small cell lung cancer [15].
- Further work is needed to assess improvement resulting from a standardised training for PD-L1 scoring of lung and other cancer types.

### What this paper adds

- This study shows that a protocol led training can improve precision in PD-L1 scoring of lung cancer specimens.
- Training either on glass or digital images show both comparable results in evaluation concordance.

## Concluding statement

This work represents an advance in biomedical science because it is the first study conducted to provide standardised training to pathologists across the world to improve precision in PD-L1 scoring of non-small cell lung specimens.

## Data Availability

The original contributions presented in the study are included in the article/Supplementary Material, further inquiries can be directed to the corresponding author.
