# Supplementary material for: Training on PD-L1 scoring in non-small cell lung cancer with high intra- and inter-reader agreement: results of a worldwide microscopic/digital image-based training of 751 pathologists
Source: Br J Biomed Sci. 2026 Jun 8;83:16477. doi: 10.3389/bjbs.2026.16477 (PMC13283929; doi:10.3389/bjbs.2026.16477)
Supplement: Supplementary file 1 [file DataSheet2.pdf]

time	1-day training	duration	time	2-day training	duration
**Day 1**			**Day 1**		
08:45 – 09:00	Welcome	15 min	08:45 – 09:00	Welcome	15 min
09:00 – 10:00	Background lecture: PD-L1 scoring of the PD-L1 22C3 ICH pharmDx in NSCLC	1 h	09:00 – 10:00	Background lecture: PD-L1 scoring of the PD-L1 22C3 IHC pharmDx in NSCLC	1 h
10:00 – 10:45	Demonstration Session: Review of PD-L1 example images Coffee break	45 min	10:00 – 10:45	Demonstration Session: Review of PD-L1 example images	45 min
10:45 – 11:00	Coffee break	15 min	10:45 – 11:00	Coffee break	15 min
11:00 – 12:00	Self-assessment: Pretest	1 h	11:00 – 12:00	Self-assessment: Pretest	1 h
12:00 – 12:30	Discussion of pretest results	30 min	12:00 – 12:30	Discussion of pretest results	30 min
12:30 – 13:15	Lunch break	45 min	12:30 – 13:15	Lunch break	45 min
13:15 – 15:30	Self-assessment: Training effectiveness test	2 h15 min	13:15 – 15:30	Self-assessment: Training effectiveness test part I	2 h15 min
15:30 – 15:45	Coffee break	15 min			
15:45 – 16:45	Discussion of self-assessment, Q&A	1 h			
16:45 – 17:30	Wrap up and certificates	45 min			
			**Day 2**		
			08:45 – 11:15	Self-assessment: Training effectiveness test part II	2 h 30 min
			11:15 – 11:30	Coffee break	15 min
			11:30 – 13:00	Review of test results and discussion of discrepant cases	1h 30 min
			13:00 – 13:45	Lunch	45 min
