# Supplementary material for: Training on PD-L1 scoring in non-small cell lung cancer with high intra- and inter-reader agreement: results of a worldwide microscopic/digital image-based training of 751 pathologists
Source: Br J Biomed Sci. 2026 Jun 8;83:16477. doi: 10.3389/bjbs.2026.16477 (PMC13283929; doi:10.3389/bjbs.2026.16477)
Supplement: Supplementary file 2 [file DataSheet1.pdf]

1-day course		2-day course			
		day 1		day 2	
case #	TPS [%]	case #	TPS [%]	case #	TPS [%]
46	0	46	0	46	0
64	0	64	0	64	0
69	0			69	0
75	0	75	0	75	0
54	1	54	1	54	1
74	5	74	5		
48	10	48	10	48	10
58	10			58	10
50	20	50	20		
53	30	53	30	53	30
63	30			63	30
71	30	71	30	71	30
73	30	73	30	73	30
52	40	52	40	52	40
62	40			62	40
47	50	47	50		
55	50	55	50	55	50
57	60			57	60
70	60			70	60
				61	70
65	70			65	70
		68	70	68	70
		56	80		
		60	80	60	80
		66	80	66	80
		49	90	49	90
		59	90		
				67	95
				72	98
		51	100	51	100

blue: cases only evaluated on day 1
orange: cases only evaluated on day 2
red: cases evaluated on day 1 and day 2
